# The Usefulness of Urinary Periostin, Cytokeratin-18, and Endoglin for Diagnosing Renal Fibrosis in Children with Congenital Obstructive Nephropathy

**DOI:** 10.3390/jcm10214899

**Published:** 2021-10-24

**Authors:** Agnieszka Turczyn, Małgorzata Pańczyk-Tomaszewska, Grażyna Krzemień, Elżbieta Górska, Urszula Demkow

**Affiliations:** 1 Department of Pediatrics and Nephrology, Medical University of Warsaw, 02-091 Warsaw, Poland; mpanczyk1@wum.edu.pl (M.P.-T.); grazyna.krzemien@wum.edu.pl (G.K.); 2 Department of Laboratory Diagnostics and Clinical Immunology of Developmental Age, Medical University of Warsaw, 02-091 Warsaw, Poland; elzbieta.gorska@uckwum.pl (E.G.); urszula.demkow@wum.edu.pl (U.D.)

**Keywords:** periostin, cytokeratin-18, endoglin, transforming growth factor-β1, renal fibrosis, congenital obstructive nephropathy, children

## Abstract

Congenital obstructive nephropathy (CON) leads to renal fibrosis and chronic kidney disease. The aim of the study was to investigate the predictive value of urinary endoglin, periostin, cytokeratin-18, and transforming growth factor-β1 (TGF-β1) for assessing the severity of renal fibrosis in 81 children with CON and 60 controls. Children were divided into three subgroups: severe, moderate scars, and borderline lesions based on 99mTc-ethylenedicysteine scintigraphy results. Periostin, periostin/Cr, and cytokeratin-18 levels were significantly higher in the study group compared to the controls. Children with severe scars had significantly higher urinary periostin/Cr levels than those with borderline lesions. In multivariate analysis, only periostin and cytokeratin-18 were independently related to the presence of severe and moderate scars, and periostin was independently related to borderline lesions. However, periostin did not differentiate advanced scars from borderline lesions. In ROC analysis, periostin and periostin/Cr demonstrated better diagnostic profiles for detection of advanced scars than TGF-β1 and cytokeratin-18 (AUC 0.849; 0.810 vs. 0.630; 0.611, respectively) and periostin for detecting borderline lesions than endoglin and periostin/Cr (AUC 0.777 vs. 0.661; 0.658, respectively). In conclusion, periostin seems to be a promising, non-invasive marker for assessing renal fibrosis in children with CON. CK-18 and TGF-β1 demonstrated low utility, and endoglin was not useful for diagnosing advanced scars.

## 1. Introduction

Congenital anomalies of the kidneys and urinary tract (CAKUT) are the leading cause of chronic kidney disease (CKD) in children, and a large part of them is related to congenital obstructive nephropathy (CON) [1,2]. The pathogenesis of CON is complex and results from various functional and morphological changes associated with impaired urinary outflow. Obstruction in the urine flow causes an increase in the pressure in the urinary tract and leads to tubular dilatation. Renal response to mechanical compression involves angiotensin (Ang)-dependent renal vasoconstriction, ischemia, hypoxia, and accumulation of reactive oxygen species (ROS) [3]. These are mediated by altered expression of growth factors, cytokines, including transforming growth factor-β1 (TGF-β1) and adhesion molecules [3,4,5]. Functional lesions lead to cell apoptosis, interstitial macrophage recruitment, epithelial–mesenchymal transition (EMT), fibroblast–myofibroblast transformation, and accumulation of extracellular matrix (ECM) proteins. Morphological changes are responsible for interstitial fibrosis, tubular and vascular atrophy, and glomerular sclerosis. CON leads to the reduction of functioning nephrons. It causes glomerular hyperfiltration and consequent glomerular sclerosis of the remaining nephrons and is responsible for the occurrence of CKD in childhood or adulthood [2,6].

Widely used imaging studies such as ultrasonography (US) and scintigraphy and currently available laboratory indices of the kidney injury such as serum creatinine (Cr), cystatin C, estimated glomerular filtration rate (GFR), and urinary albumin excretion do not provide information about the early stage of renal fibrosis and are poor predictors of the future course of CON [2,7]. Therefore, unfavorable prognosis in children with CON mandates a search for non-invasive fibrotic markers, which would enable early diagnosis of the fibrosis process, assessment of the severity of fibrosis, and early intervention in children at risk of renal injury progression.

TGF-β1 is a 25 kDA well-known cytokine that plays a pivotal role in renal fibrosis. It is secreted by epithelial cells, macrophages, and fibroblasts as an inactive latent complex [5,8]. After activation, TGF-β1 binds to its type II receptor (TβRII), which recruits two types I receptors (TβRI)—Activin like kinase 1 (ALK1) and ALK5. Depending on the TβRI recruited, different kinds of Smad proteins become phosphorylated for cellular response to TGF-β1. The complex composed of TβRII and ALK1 leads to Smad1/5/8 phosphorylation, and the complex composed of TβRII and ALK5 leads to Smad2/3 phosphorylation. Smad cascade is one of the most essential and well-known pathways in progressive renal fibrosis [9,10,11]. TGF-β1 promotes fibrosis by cell apoptosis, increasing macrophage infiltration, EMT, and myofibroblast transformation [3]. Overexpression of TGF-β1 mRNA and protein was demonstrated in multiple models of renal fibrosis [5,12,13]. Increased urinary TGF-β1 levels were found in CKD [14] and various types of nephropathies, including glomerular (GN), diabetic (DN), and obstructive nephropathy [8,15,16,17].

Endoglin is a 180 kDa homodimeric protein consisting of extracellular, transmembrane, and intracellular domain. Endoglin expression was identified in endothelial, smooth muscle and mesangial cells, macrophages, and fibroblasts [9,18,19]. Ang II and TGF-β1 are the most important stimulators of endoglin expression [18,20]. Endoglin acts as a TGF-β1 co-receptor known as TβRIII and binds to TGF-β1 by ALK1 and ALK5 receptors [18]. It modulates cellular responses to TGF-β1, mainly related to myofibroblast transformation and ECM accumulation [11,20]. Cytoplasmatic domain differentiates two endoglin isoforms, named large (L-Eng) and short (S-Eng), according to the length of their cytoplasmatic tails. Upregulation of L-Eng leads to Smad1 and Smad2/3 phosphorylation and promotes fibrosis, while upregulation of S-Eng acts oppositely [9,19]. Overexpression of endoglin was shown in renal fibrosis models [21,22,23] and in various human diseases, including CKD and DN [11,24].

Periostin is a 90 kDa matricellular protein with a multi-domain structure [25,26,27]. It is induced during organogenesis and found only in small amounts in a healthy kidney. Periostin was expressed de novo during the chronic disease of several organs [26,28,29]. In the kidney, periostin is released from fibroblasts and myofibroblasts in response to many factors, including Ang II and TGF-β1, which stimulate periostin expression by Smad phosphorylation and through Smad independent pathways [25,30]. It is expressed mainly in the location of tissue damage in ECM areas and directly interacts with multiple ECM proteins such as integrin, fibronectin, collagens, tenascin C, and bone morphogenic protein-1 (BMP-1) [27,28]. After binding to integrin, periostin induces fibrosis by increasing inflammatory cell infiltration, ECM remodeling, and TGF-β1 pathway upregulation [29,31]. Overexpression of periostin was reported in numerous experimental and clinical studies of renal fibrosis [25,32]. Increased urinary periostin levels were described in CKD, polycystic kidney disease (PKD), and various types of nephropathies, including IgA nephropathy (IgAN) [29,32,33].

Cytokeratin-18 (CK-18) is a 45 kDA cytoskeletal protein consisting of three domains [34,35]. It is a family member of intermediate filament proteins and represents about 5% of total cell proteins in most epithelial and parenchymal cells [36,37]. CK-18 is involved in maintaining cell shape and integrity, mechanical stability, intracellular organization, and cell signaling, transport, and differentiation [35,38,39]. In addition, it protects cells from mechanical and non-mechanical stress [35,40]. In response to various stress situations, CK-18 undergoes phosphorylation that modifies its solubility, filaments organization, prevents interaction with other molecules, and protects from proteolysis and degradation [35,39]. Overexpression of CK-18 was found in models of renal fibrosis and in humans with diabetic and lupus nephropathy [39]. High urinary CK-18 levels were reported in acute kidney injury (AKI) and CKD [37,39].

To the best of our knowledge, there are no studies in the available literature investigating the usefulness of urinary endoglin, periostin, and CK-18 in children with CON. Therefore, the aim of our study was to investigate the predictive value of urinary endoglin, periostin, CK-18, and TGF-β1 for assessing the severity of renal fibrosis in children with congenital obstructive nephropathy.

## 2. Materials and Methods

### 2.1. Study Group

**Figure 1:**
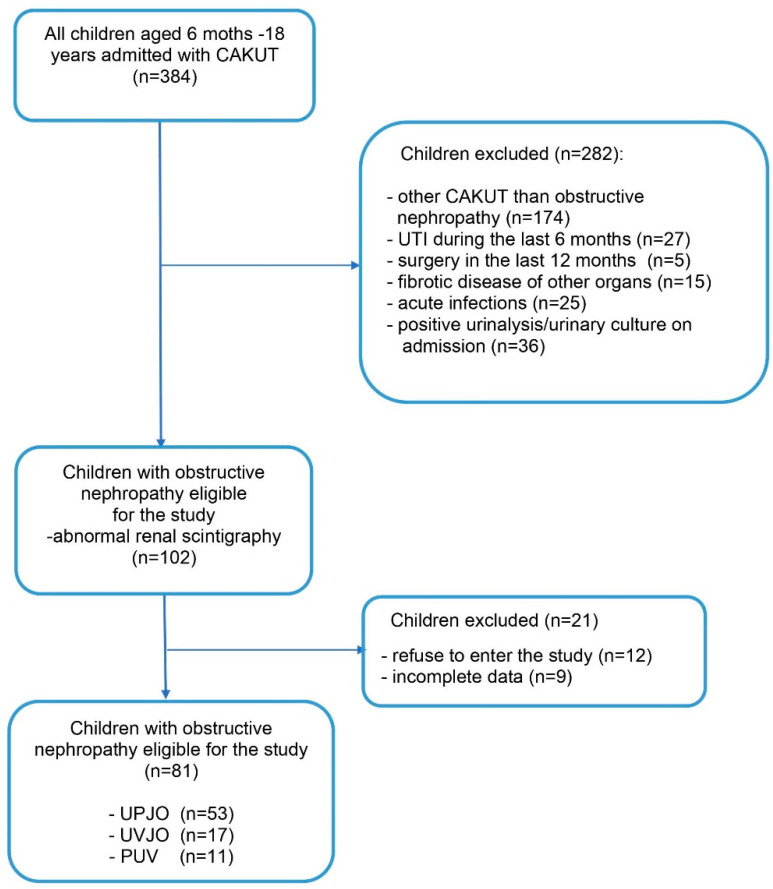
Study flow diagram. CAKUT—congenital anomalies of the kidney and urinary tract; UTI—urinary tract infection; UPJO—ureteropelvic junction obstruction; UVJO—ureterovesical junction obstruction; PUV—posterior urethral valves.

This single-center cross-sectional study included 81 children with congenital obstructive nephropathy who were diagnosed at the Department of Pediatrics and Nephrology of the Medical University of Warsaw, Poland. The study was performed from July 2016 to April 2019. The inclusion criteria were age from 6 months to 18 years and presence of obstructive nephropathy: ureteropelvic junction obstruction (UPJO), ureterovesical junction obstruction (UVJO), and posterior urethral valves (PUV). The exclusion criteria were occurrence of another CAKUT history of urinary tract infection (UTI) during the last 6 months, surgery in the last 12 months, fibrotic disease of other organs, and acute infections (temporary exclusion). Sixty healthy children were included in the control group. The study design is displayed on the flow diagram in Figure 1. A sample size of the study and control groups was estimated based on the available literature on renal biomarkers with a statistical power of 0.8 and statistical significance *p* = 0.05.

### 2.2. Ethical Issues 

The study was approved by the local Bioethics Committee for Human Research (No. KB/152/2016) before the initiation. The clinical investigation was conducted in accordance ethical standards of the institutional research committee and with the guidelines of the 2013 Declaration of Helsinki. Informed consent was obtained from all participant representatives and participants (≥16 years) prior to study inclusion.

### 2.3. Clinical Parameters

In all participants, the following clinical data were evaluated: age (years), sex, body height (cm), body weight (kg), and blood pressure measurement.

### 2.4. Biochemical Parameters

In all participants, the following laboratory data were evaluated: serum Cr (mg/dL), serum cystatin C (ng/L), urinary albumin (mg/L), and urinary Cr (mg/dL). Urinalysis and urine culture were performed to rule out urinary tract infection (UTI). Estimated GFR (mL/min/1.73 m^2^), according to the revised 2009 Schwartz formula [41], and urinary albumin to urinary Cr ratio (ACR) were calculated. Biochemical parameters were measured using VITROS 5600 Integrated System (Ortho Clinical Diagnostics) except cystatin C (IMMAGE 800 Beckman Coulter Company, Koto, Tokyo). In line with local recommendations, normal serum Cr (depending on age 0.2–0.7 mg/dL), serum cystatin C (depending on age 0.51–1.15 mg/L), and ACR (<30 mg/g Cr) were derived from the manufacturers′ reference values.

### 2.5. Urinary Biomarkers

Urine samples were taken on fasting in the morning, immediately centrifuged according to the manufacturers′ instructions, frozen, and stored at −80 °C until further assays were performed. All biomarkers were measured using enzyme-linked immunosorbent assays (ELISA) kits from Reagent Genie, Dublin, Ireland: TGF-β1-Cat. No. HUFI 00248, Human TGF-β1/TGF-beta-1; endoglin-Cat. No. HUFI 00064, Human CD105/Endoglin; periostin-Cat. No. HUDLO 0236 Human Periostin (POSTN); cytokeratin-18-Cat. No. HUFI 02320, Human CK-18/KRT18/Cytokeratin-18. The urinary TGF-β1,endoglin, periostin and CK-18 levels were normalized to the urinary Cr levels, measured from the same urine samples, and expressed as ng/mg Cr.

### 2.6. Imaging Data

In all patients, abdominal ultrasonography and 99mTechnetium-ethylenedicysteine (99mTc-EC) scintigraphy were evaluated at the study entry. US was performed using Philips Epiq 5G device (Royal Philips, Amsterdam, The Netherlands) in B-mode. Pelvic dilatation was classified based on the anterior–posterior diameter (mm) (APD) as follows: severe APD—>20 mm, moderate APD—10–20 mm, and mild APD—<10 mm [42,43].

99mTc-EC was conducted to evaluate renal scarring and relative renal function (RRF). Children were injected with an adequate dose of 99mTc-EC for the child′s age and body weight. Two experienced nuclear medicine specialists evaluated radionuclide scans. Renal scars were diagnosed as an abnormal parenchymal layer, irregular renal contour, disorders of tracer placement and uptake [44]. RRF of affected kidney ≥45% was considered normal, and RRF of <45% or >55% was considered abnormal. Renal scars were classified as follows: severe—narrowed parenchymal layer, focal or diffuse tracers placement disorders, and renal radionuclide uptake below 45%; moderate—narrowed parenchymal layer or abnormal kidney outline, focal or diffuse tracers placement disorder, and renal radionuclide uptake above or equal to 45%; borderline lesions—normal parenchymal layer, discreet tracers placement disorder and renal radionuclide uptake above or equal to 45%.

Voiding cystourethrography (VCUG) was evaluated at the moment of CON diagnosis. Children with VUR were excluded from the study, except those with secondary VUR as a consequence of PUV.

### 2.7. Statistical Analysis

Statistical analysis was performed using Statistica 13.3 PL software (StatSoft, Tulsa, OK, USA). The results were presented as the mean ± standard deviation or the median, interquartile ranges based on the Shapiro–Wilk and the Lilliefors normality test results. The Student′s *t*-test or the Mann–Whitney U-test was used to compare two groups of variables. ANOVA test or Kruskal–Wallis test and post hoc test were performed to analyze differences between three subgroups. The relationship between variables was evaluated using the Pearson or the Spearman′s rank correlation. Odds ratio (OR), including 95% confidence interval (CI), were calculated by univariate and multivariate logistic regression analysis to identify variables associated with the presence of renal scars. Variables associated with renal scars in the univariate analysis were included in the multivariate model. Variables that correlated with each other with r > 0.600 were excluded from the regression model to avoid collinearity. Receiver operating curve (ROC) analysis was used to calculate the area under the curves (AUC) for laboratory variables and to find the best cut-off values (including 95% CI), sensitivity, and specificity in the detection of renal scarring for each variable. *p* values less than 0.05 were considered statistically significant for all tests.

## 3. Results

### 3.1. Clinical and Laboratory Parameters

Clinical and laboratory data in the study group and in the control group are shown in Table 1. We evaluated 81 children with CON and 60 healthy children. CON was diagnosed due to UPJO in 53 (65%) of children, UVJO in 17 (21%) of children, and PUV in 11 (14%) of children. US at the moment of study entry found severe dilatation of pelvis in 23% of children, moderate dilatation in 63% of children, and mild in 14% of children.

Children with CON had significantly higher levels of cystatin C and ACR, significantly lower GFR, and significantly higher urinary periostin, CK-18, and periostin/Cr compared to the controls. Groups did not differ in terms of age, sex, serum Cr and other urinary biomarkers. The levels of serum Cr were slightly elevated in 6 (7.4%) children, cystatin C in 15 (18.5%) children, and ACR in 15 (18.5%) children. Arterial hypertension was diagnosed in four children who were treated with calcium channel blocker (three children) and angiotensin-converting enzyme inhibitor (one child). Fourteen (17%) children were treated surgically because of a significant obstruction in urine outflow.

### 3.2. Renal Scintigraphy

Comparison of laboratory variables with the results of renal scintigraphy in the study group is shown in Table 2. According to the 99mTc-EC results, children with CON were divided into three subgroups: group 1 (n = 32, 39.5%)—severe renal scars, group 2 (n = 31, 38.3%)—moderate renal scars, and group 3 (n = 18, 22.2%)—borderline lesions. Children with severe scars had a significantly higher periostin/Cr ratio than those with borderline results. No other differences in laboratory indices of kidney injury and urinary biomarkers were found between children with severe, moderate scars, and borderline lesions. The group of borderline lesions demonstrated significantly higher levels of cystatin C and urinary periostin compared to the control group (0.92 ± 0.21 vs. 0.81 ± 0.15, *p* = 0.012; 0.102 (0.034; 0.149) vs. 0.028 (0.013; 0.053) *p* = 0.001, respectively) and significantly lower levels of GFR compared to the control group (106.18 ± 19.11 vs. 121.60 ± 26.09, *p* = 0.018). The groups did not differ in terms of other laboratory indices of kidney injury and urinary biomarkers (data not shown).

Normal RRF (45–55%) demonstrated 49 (60.5%) of children and decreased RRF < 45% 32 (39.5%) of children. RRF less than 10% was found in 2 (2.5%) of children, RRF 10–19% in 4 (5%) of children, 20–39% in 13 (16%) of children, and 40–44% in 13 (16%) of children. Correlations of urinary biomarkers with RRF in the scintigraphy in the study group are shown in Table 3. No correlation between urinary biomarkers and RRF was found.

### 3.3. Correlation of Laboratory Parameters

Correlations between urinary biomarkers and widely used laboratory indices of kidney injury in the studied children are shown in Table 4. All standardized to Cr biomarkers correlated with cystatin C and ACR. There was a negative correlation between biomarkers and serum Cr. None of the biomarkers correlated with GFR except endoglin/Cr.

Inter-correlations of normalized urinary biomarkers in the studied children are shown in Table 5. A strong positive correlation was found between TGF-β1/Cr and endoglin/Cr, TGF-β1/Cr, and CK-18/Cr, as well between endoglin/Cr and CK-18/Cr. A weaker correlation was revealed between periostin/Cr and other biomarkers.

### 3.4. Relation between Laboratory Parameters and Renal Scars

Univariate and multivariate logistic regression analysis of laboratory variables related to the presence of severe and moderate scars are shown in Table 6a, and related to the presence of borderline lesions are shown in Table 6b. In univariate logistic regression analysis, cystatin C, GFR ACR, and urinary biomarkers—TGF-β1, CK-18, periostin, and periostin/Cr were associated with the presence of severe and moderate renal scars. Multivariate analysis demonstrated that GFR, TGF-β1, and periostin were independently related to the presence of severe and moderate scars. Urinary periostin/Cr was excluded from the multivariate model due to collinearity.

Cystatin C, GFR, periostin, and endoglin/Cr were related to the presence of borderline lesions in univariate analysis, whereas in multivariate analysis, cystatin C and periostin were independently related to borderline lesions. Endoglin/Cr was excluded from multivariate analysis due to collinearity.

Diagnostic usefulness of laboratory variables for diagnosing severe and moderate renal scars in the scintigraphy is shown in Table 7a, and for diagnosing borderline lesions, in the scintigraphy in Table 7b. ROC analysis demonstrated that periostin and periostin/Cr had higher AUC for detection of severe and moderate scars than cystatin C, GFR, ACR, TGF-β1, and CK-18 (Table 7a), and periostin had higher AUC than cystatin C, GFR, endoglin, and periostin/Cr for diagnosing borderline lesions (Table 7b).

## 4. Discussion

Our cross-sectional single-center study investigated the predictive value of new biomarkers, such as endoglin, periostin, and CK-18, for assessing the severity of renal scarring in children with CON. We also evaluated TGF-β1, the most recognized fibrotic factor, which served as our reference (“gold standard”). Here, we report that urinary periostin, periostin/Cr, and CK-18 were significantly higher in children with CON compared to the healthy controls. In addition, children with severe scars had significantly higher urinary periostin/Cr levels than those with borderline lesions. Thus, periostin and periostin/Cr demonstrated a better diagnostic profile for diagnosing renal scars than other evaluated biomarkers. However, both periostin and periostin/Cr did not differentiate severe and moderate scars from borderline lesions. Endoglin showed moderate usefulness for diagnosing borderline lesions, and CK-18 and TGF-β1 revealed low utility for diagnosing severe and moderate scars.

Multiple clinical studies confirmed the usefulness of urinary TGF-β1 for the diagnosis of urinary tract obstruction and tubulointerstitial fibrosis. Children with obstructive uropathy demonstrated significantly higher TGF-β1 levels than those with non-obstructive hydronephrosis and healthy controls [4,45,46]. A significant decrease in elevated TGF-β1 levels was observed after pyeloplasty [47]. Patients who improved renal function after nephrostomy (i.e., those in whom GFR increased) had significantly lower TGF-β1 levels at presentation compared to those who did not [48]. Elevated urinary TGF-β1 demonstrated 82% accuracy for differentiation reversible obstruction from irreversible [48] and 90.8% accuracy for long-term follow-up after pyeloplasty [47]. The ESCAPE trial revealed markedly higher urinary TGF-β1 levels in children with mild to moderate CKD and CON compared to those with CKD and other kidney diseases [14].

In contrast, our study found no differences in urinary TGF-β1 and TGF-β1/Cr levels between children with CON and healthy controls. Palmer et al. reported elevated TGF-β1 levels only in the pelvis of the obstructed kidney but not in the bladder urine [49]. Some authors suppose that the production of TGF-β1 is increased in the early phase of tissue damage and reduced in the terminal stage of tissue degeneration [14,17]. We did not find any differences in TGF-β1 and TGF-β1/Cr levels between subgroups with severe, moderate scars and borderline lesions. Despite this, urinary TGF-β1 was associated with the presence of advanced scars. However, its diagnostic potential for detecting scars was low. In our study, 17% of children were at least one year after surgery of urinary tract obstruction, which could reduce their TGF-β1 level. A slight increase in serum Cr was found in 7.4% of children and in cystatin C in 18.5% of children. RRF in scintigraphy less than 10% demonstrated in 2.5% of patients. It could also have had an impact on the results obtained.

The importance of endoglin in renal fibrosis was documented in several animal models of renal fibrosis. For example, the model of tubulointerstitial fibrosis induced by unilateral ureteral obstruction (UUO) demonstrated an increase in endoglin mRNA and protein expression in the obstructed kidney [9,19,50]. Model of renal ischemia–reperfusion (I-R) injury showed coincidence endoglin expression with increased TGF-β1 mRNA expression. In this study, haploinsufficient mice of endoglin (Eng+/−) were protected for renal I-R injury compared to their wild type (WT) littermates (Eng+/+) [20]. Roy-Chaudhury et al. described an association of interstitial endoglin expression and chronic histological damage in biopsies from patients with progressive CKD [24]. A recent study by Gerrits et al. revealed an increase in interstitial endoglin expression in autopsy samples obtained from diabetic patients with DN compared to those without DN. Renal endoglin expression correlated with the degree of interstitial fibrosis and increased serum Cr, reduced GFR, and hypertension in DN [11].

In our cohort of pediatric patients, we demonstrated no differences in urinary endoglin and endoglin/Cr levels between children with CON and the controls, as well as between subgroups with severe, moderate scars, and borderline lesions. Only endoglin/Cr was related to the presence of borderline lesions. In ROC analysis, endoglin showed moderate utility as a biomarker for diagnosing borderline lesions. However, its specificity was very high. We did not evaluate L-Eng and S-Eng isoforms. Therefore, we do not know if an association of endoglin with borderline lesions is due to the upregulation of pro-fibrotic L-Eng or anti-fibrotic S-Eng, especially in children with borderline lesions.

Recent studies have identified periostin as a novel key factor in the progression of kidney disease. Overexpression of periostin following renal injury was described in different models of renal fibrosis, such as UUO, 5/6 nephrectomy, hypertensive renal injury, and renal I-R injury [31,51,52]. High expression of periostin was associated with acceleration of cyst growth and fibrosis in PKD [33,53]. Periostin overexpression was observed in patients with different types of progressive nephropathies, including lupus nephropathy and chronic allograft nephropathy (CAN) [7,32,54]. Patients with high levels of urinary periostin at the time of AKI episode were more likely to progress to CKD [52]. In the study by Hwang et al., higher urinary periostin/Cr levels at the time of renal biopsy were associated with tissue periostin overexpression, a higher degree of fibrosis, a greater decline in GFR, and poor renal outcome in IgAN patients [29]. Some studies demonstrated a protective effect of periostin suppression for CKD progression. Periostin-null mice showed a decrease in apoptosis compared to the WT mice [25,52].

In the present study, we demonstrated significantly higher urinary periostin and periostin/Cr levels in children with CON compared to the controls and significantly higher periostin/Cr levels in children with severe scars compared to those with borderline lesions. Both periostin and periostin/Cr demonstrated relatively high utility for diagnosing scars and moderate utility for diagnosing borderline lesions. However, based on diagnostic profile and cut-off in ROC analysis, periostin did not allow for differentiation of severe and moderate scars from borderline lesions.

It is difficult to explain why periostin was of no use to differentiate scars from borderline lesions, especially that we showed significantly higher levels of periostin/Cr in children with severe scars compared to those with borderline lesions. Although we confirmed that periostin is strongly related to renal fibrosis, we still know too little about the regulation of periostin expression and its signaling pathways [33]. Recent evidence suggests that periostin might be a tissue repair molecule that stimulates signaling pathways involved in tissue regeneration [33,55,56]. Korman et al. revealed a protective effect of periostin in the model of AKI induced by renal I-R injury. They demonstrated that periostin overexpression was associated with lower expression of pro-inflammatory cytokines, less epithelial damage, increased proliferation of pro-regenerative macrophage phenotypes, and less severe injury than the periostin-null mouse. In the authors′ opinion, periostin may play a protective role in AKI and a detrimental role in CKD [56]. In our study, we followed patients with chronic renal fibrosis. Therefore, periostin overexpression could not be related to renal tissue regeneration.

CK-18 is a cell-protective and stress-responsive protein that seems to be a novel sensitive marker of renal tubular cell stress and tubular injury. Experimental studies demonstrated an increase in CK-18 expression in different models of renal injury. In the model of UUO, upregulation of CK-18 occurred very early after UUO induction and increased with renal injury progression. Additionally, the model of progressive GN with secondary tubulointerstitial injury and fibrosis showed only slight CK-18 expression in the early stage of disease and significantly higher expression in the late stage. Overexpression of CK-18 in renal biopsy was reported in the majority of renal tubules in the cast, diabetic, and lupus nephropathy, as well in the Bowman capsule in crescentic necrotic GN [39].

In this study, we found significantly higher urinary CK-18 levels in children with CON compared to the controls, but no differences were observed in terms of CK-18 and CK-18/Cr between the three subgroups. Urinary CK-18 was associated with the presence of scars, but its diagnostic value was low. In the study by Djudjaj et al., elevated urinary CK-18 levels were found in animals with adenine nephropathy and Alport syndrome and in patients with AKI [39]. Roth et al. reported significantly higher urinary CK-18 levels in CKD stage 5 compared to the healthy controls [37]. During cell degeneration, CK-18 is released in two forms depending on the type of cell death. In cells apoptosis, caspase cleaved CK-18 fragments are produced, while in cell necrosis, CK-18 is liberated without caspase-mediated modifications [57]. In CKD, cell loss results mainly from cell necrosis, while in the obstructed kidney, cell death is most susceptible to apoptosis [3,58]. Choi et al. confirmed a progressive increase in tubular and interstitial cell apoptosis during the duration of renal obstruction [59]. We did not assess caspase cleaved CK-18. However, we can speculate that our patients could have high levels of CK-18 fragments due to apoptosis followed by urinary tract obstruction.

It is not known whether urinary fibrotic biomarkers are associated with clinical and laboratory indices of kidney injury. In the ESCAPE trial, GFR inversely correlated with urinary TGF-β1 [14]. Similarly, a negative correlation was reported with urinary periostin in IgAN [29] and chronic allograft nephropathy (CAN) [7] and urinary CK-18 in CKD [37]. Some studies reported positive correlations of TGF-β1 with proteinuria and urinary α1-microglobulin in CON [4], urinary periostin with proteinuria in CAN [7], and CK-18 with proteinuria and albuminuria in CKD stage 5 [37]. In contrast, other authors did not show any correlations of urinary TGF-β1 with serum Cr, GFR, ACR, proteinuria, and degree of tubulointerstitial fibrosis [4,17].

We found positive correlations of the normalized biomarkers with cystatin C and ACR, negative correlations with serum Cr, and no relation with GFR except endoglin/Cr. These negative correlations with serum Cr were probably related to our study′s relatively large number of young children. In infants and young children, low serum Cr levels, due to small muscle mass in this age, and greater Cr tubular reabsorption lead to low urinary Cr excretion [2]. It results in higher values of normalized biomarkers and is responsible for a negative correlation with serum Cr. Due to the heterogenicity of children′s age in our study, we assessed biomarkers with and without normalization.

In our study, we also evaluated the association of widely used laboratory indices of kidney injury with the presence of renal scars and borderline lesions. We found that only ACR was able to differentiate children with scars from those with borderline lesions. However, its diagnostic value was relatively low. The ESCAPE trial also demonstrated inter-correlation of urinary TGF-β1 with other biomarkers that participated in the progression of tissue injury in CKD [14]. In line with this study, we found an inter-correlation of all normalized biomarkers. These results are not unexpected. It is well-known that TGF-β1 is the major stimulator of endoglin and periostin expression, periostin creates a feedback loop with TGF-β1, and CK18 was identified as an important factor in renal fibrosis [9,10,27,32,33,39].

Some studies evaluated the correlation of the intensity of renal fibrosis and urinary biomarkers with RRF of obstructed kidney in a nuclear renal scan. Zhang demonstrated a correlation between the intensity of renal fibrosis in renal biopsy performed during pyeloplasty and RRF of affected kidney [60]. In contrast, other authors did not show any correlation between urinary TGF-β1 and RRF [4,47]. In the present study, 99mTc-EC scintigraphy was used for renal scars assessment, which is an alternative to 99mTc-dimercaptosuccinic acid (DMSA) scintigraphy, with high sensitivity (98.75%) and specificity (99.15%) for detection of scars in normally positioned kidneys but with lower radiation dose [61]. We demonstrated that approximately half of our children with advanced scars demonstrated tracer uptake higher than 45%. We also did not find a correlation between urinary biomarkers and RRF in children with CON.

Urinary tract obstruction leads to a reduction in functioning nephrons and to the compensatory adaptation of the remaining. Therefore, it may affect RRF results [2]. In addition, in hydronephrosis, tracer accumulation in the obstructed kidney, as well as an increase in blood flow caused by altered renal hemodynamics, may cause falsely high RRF [62,63]. Men-Meir et al. demonstrated that “supranormal” RRF in the obstructed kidney is not always a favorable sign and may even be a warning sign of impending decompensation. Therefore, a measure of RRT has a low predictive value for renal fibrosis in children with CON.

Our study has some limitations. This single-center research may be associated with the bias occurrence. The cross-sectional design of the study prevents from drawing definitive and causative conclusions. The number of analyzed patients in subgroups was relatively low. Heterogenicity of patients′ ages, various causes of obstructive nephropathy, and different patient follow-up may impact the results. In addition, we have not analyzed the fibrosis markers in other groups of patients, e.g., in glomerulopathies.

## 5. Conclusions

To the best of our knowledge, this study, for the first time, demonstrates that periostin, endoglin, and CK-18 are associated with renal fibrosis in children with congenital obstructive nephropathy. Periostin showed a higher diagnostic profile for diagnosing renal scars than other evaluated biomarkers. However, periostin did not differentiate advanced scars from borderline lesions. CK-18 and TGF-β1 demonstrated low utility, and endoglin was not useful for diagnosing advanced scars. In our opinion, periostin seems to be a promising, non-invasive marker for the assessment of renal fibrosis in children with CON. However, future studies on more patients are needed to confirm our results.

## Figures and Tables

**Table 1:** Clinical and laboratory data in the study group and in the control group.

	Study Group	Control Group	*p*
Variables	(*n* = 81)	(*n* = 60)	
Demographic data			
Sex, male, *n* (%)	58 (71.6)	39 (65.0)	0.223
Age at the diagnosis (years)	0.40 (0.10; 4.98)		-
Age at the study entry (years)	3.92 (0.67; 9.17)	4.08 (1.83; 10.08)	0.247
Obstructive uropathy, *n* (%)			
UPJO	53 (65)		
UVJO	17 (21)		
PUV	11 (14)		
Ultrasonography			
Dilatation of renal pelvis, *n* (%)			
Severe (APD > 20 mm)	19 (23)		
Moderate (APD 10–20 mm)	51 (63)		
Mild (APD < 10 mm)	11 (14)		
Laboratory data			
Serum Cr (mg/dL)	0.40 (0.30; 0.50)	0.40 (0.30; 0.50)	0.603
Cystatin C (mg/L)	0.91 ± 0.22	0.81 ± 0.15	0.005
GFR (mL/min/1.73 m^2^)	106.90 ± 18.57	121.60 ± 26.09	<0.001
ACR (mg/g)	16.87 (7.04; 26.12)	11.67 (4.85; 18.83)	0.024
Urinary biomarkers			
TGF-β1 (ng/mL)	0.038 ± 0.021	0.046 ± 0.027	0.069
Endoglin (ng/mL)	8.82 ± 3.68	9.85 ± 4.86	0.090
Periostin (ng/mL)	0.092 (0.062; 0.160)	0.028 (0.013; 0.053)	<0.001
Cytokeratin-18 (ng/mL)	0.383 ± 0.152	0.320 ± 0.162	0.012
TGF-β1/Cr (ng/mg)	0.088 (0.028; 0.261)	0.103 (0.040; 0.281)	0.450
Endoglin/Cr (ng/mg)	21.39 (6.55; 57.04)	22.47 (9.82; 55.55)	0.580
Periostea/Cr (ng/mg)	0.258 (0.131; 0.508)	0.081 (0.024; 0.164)	<0.001
Cytokeratin-18/Cr (ng/mg)	0.838 (0.310; 1.984)	0.562 (0.293; 1.580)	0.260

UPJO—ureteropelvic junction obstruction; UVJO—ureterovesical junction obstruction; PUV—posterior urethral valves; APD—anterior–posterior diameter; Cr—creatinine; GFR—estimated glomerular filtration rate; ACR—urinary albumin/creatinine ratio; TGF-β1—transforming growth factor- β1.

**Table 2:** Comparison of laboratory variables with the results of renal scintigraphy in the study group.

Variables	Severe Scars	Moderate Scars	Borderline Lesions
	(1)	(2)	(3)
**No. patients (%)**	32 (39.5%)	31 (38.3%)	18 (22.2%)
Laboratory data			
Serum Cr (mg/dL)	0.35 (0.3; 0.5)	0.3 (0.3; 0.5)	0.5 (0.33; 0.6)
Cystatin C (mg/L)	0.90 ± 0.22	0.91 ± 0.23	0.92 ± 0.21
GFR (mL/min/1.73 m^2^)	107.47 ± 21.37	106.70 ± 15.5	106.18 ± 19.11
ACR (mg/g)	17.22 (7.31; 25.00)	19.2 (9.06; 26.74)	9.45 (4.09; 24.28)
Urinary biomarkers			
TGF-β1 (ng/mL)	0.039 ± 0.018	0.036 ± 0.020	0.040 ± 0.026
Endoglin (ng/mL)	9.07 ± 4.57	9.22 ± 3.25	7.70 ± 2.31
Periostin (ng/mL)	0.098 (0.073; 0.208)	0.080 (0.063; 0.125)	0.102 (0.034; 0.149)
Cytokeratin-18 (ng/mL)	0.370 ± 0.142	0.391 ± 0.153	0.394 ± 0.174
TGF-β1/Cr (ng/mg)	0.095 (0.034; 0.286)	0.088 (0.026; 0.238)	0.038 (0.014; 0.228)
Endoglin/Cr (ng/mg)	22.16 (8.30; 60.55)	25.16 (8.65; 62.36)	9.73 (4.48; 44.45)
Periostin/Cr (ng/mg)	0.342 (0.161; 0.616) *	0.269 (0.172; 0.473)	0.110 (0.070; 0.250)
Cytokeratin-18/Cr (ng/mg)	0.976 (0.355; 2.188)	0.961 (0.530; 1.774)	0.442 (0.268; 1.530)

Cr—creatinine; GFR—estimated glomerular filtration rate; ACR—urinary albumin/creatinine ratio; TGF-β1—transforming growth factor-β1. * *p*—0.010 versus borderline lesions.

**Table 3:** Correlations between urinary biomarkers and %RRF in the study group.

Variables	%RRF	
	r	*p*
TGF-β1	0.04	0.708
Endoglin	0.10	0.386
Periostin	−0.15	0.190
Cytokeratin-18	−0.01	0.921
TGF-β1/Cr	0.03	0.785
Endoglin/Cr	0.07	0.560
Periostin/Cr	−0.12	0.293
Cytokeratin-18/Cr	0.02	0.868

%RRF—relative renal function Cr—creatinine; TGF-β1—transforming growth factor-β1.

**Table 4:** Correlations between urinary biomarkers and laboratory indices of kidney injury in the studied children.

Variables	Serum Cr		Cystatin C		GFR		ACR	
	r	*p*	r	*p*	r	*p*	r	*p*
TGF-β1/Cr	−0.45	<0.001	0.27	<0.001	0.14	0.104	0.35	<0.001
Endoglin/Cr	−0.54	<0.001	0.24	0.004	0.18	0.037	0.44	<0.001
Periostin/Cr	−0.48	<0.001	0.26	0.002	0.11	0.210	0.49	<0.001
Cytokeratin-18/Cr	−0.57	<0.001	0.27	<0.001	0.06	0.448	0.47	<0.001

Cr—creatinine; GFR—estimated glomerular filtration rate; ACR—urinary albumin/creatinine ratio; TGF-β1—transforming growth factor-β1.

**Table 5:** Inter-correlations of normalized urinary biomarkers in the studied children.

Variables	TGF-β1/Cr		Periostin/Cr		Cytokeratin-18/Cr	
	r	*p*	r	*p*	r	*p*
TGF-β1/Cr	-	-	0.37	<0.001	0.71	<0.001
Endoglin/Cr	0.72	<0.001	0.53	<0.001	0.73	<0.001
Periostin/Cr	0.39	<0.001	-	-	0.54	<0.001

TGF-β1—transforming growth factor-β1.

**Table 6:** Univariate and multivariate logistic regression analysis of laboratory variables related to the presence of severe, moderate renal scars and borderline lesions in the scintigraphy. **a.** Univariate and multivariate logistic regression analysis of laboratory variables related to the presence of severe and moderate renal scars in the scintigraphy (*n* = 63). **b.** Univariate and multivariate logistic regression analysis of laboratory variables related to the presence of borderline lesions in the scintigraphy (*n* = 18).

Variables	Univariate Regression Analysis		Multivariate Regression Analysis	
	OR (95% CI)	*p*	OR (95% CI)	*p*
**a**				
**Laboratory data**				
Serum Cr (mg/dL)	1.23 (0.15–9.90)	0.841	-	-
Cystatin C (mg/L)	12.58 (1.62–97.58)	0.014	7.09 (0.61–82.54)	0.114
GFR (mL/min/1.73 m^2^)	0.97 (0.95–0.99)	0.001	0.96 (0.94–0.98)	<0.001
ACR (mg/g)	1.03 (1.00–1.06)	0.027	1.04 (1.00–1.07)	0.053
**Urinary biomarkers**				
TGF-β1 (ng/mL)	0.85 (0.72–0.99)	0.036	0.89 (0.73–1.08)	0.225
Endoglin (ng/mL)	0.96 (0.89–1.05)	0.374	-	-
Periostin (ng/mL)	1.28 (1.15–1.43)	<0.001	1.26 (1.12–1.40)	<0.001
Cytokeratin-18 (ng/mL)	12.93 (1.13–148.19)	0.038	25.99 (1.25–539.35)	0.033
TGF-β1/Cr (ng/mg)	0.99 (0.98–1.01)	0.441	-	-
Endoglin/Cr (ng/mg)	0.90 (0.65–1.24)	0.512	-	-
Periostin/Cr (ng/mg) *	1.05 (1.03–1.07)	<0.001	-	-
Cytokeratin-18/Cr (ng/mg)	1.13 (0.92–1.39)	0.229	-	-
**b**				
**Laboratory data**				
Serum Cr (mg/dL)	14.23 (0.75–269.34)	0.072	-	-
Cystatin C (mg/L)	35.80 (1.45–885.94)	0.026	62.83 (1.65–2397.05)	0.023
GFR (mL/min/1.73 m^2^)	0.97 (0.94–1.00)	0.027	1.00 (0.96–1.04)	0.055
ACR (mg/g)	1.00 (0.96–1.04)	0.906	-	-
**Urinary biomarkers**				
TGF-β1 (ng/mL)	0.91 (0.75–1.11)	0.343	-	-
Endoglin (ng/mL)	0.89 (0.78–1.01)	0.077	-	-
Periostin (ng/mL)	1.20 (1.08–1.33)	0.001	1.17 (1.05–1.31)	0.005
Cytokeratin-18 (ng/mL)	14.48 (0.55–384.17)	0.104	-	-
TGF-β1/Cr (ng/mg)	1.00 (0.98–1.02)	0.939	-	-
Endoglin/Cr (ng/mg) **	0.61 (0.38–0.98)	0.038	-	-
Periostin/Cr (ng/mg)	1.01 (0.99–1.04)	0.288	-	-
Cytokeratin–18/Cr (ng/mg)	0.95 (0.66–1.38)	0.805	-	-

Cr—creatinine; GFR—estimated glomerular filtration rate; ACR—urinary albumin/creatinine ratio; TGF-β1—transforming growth factor-β1. * Periostin/Cr excluded from multivariate model due to collinearity. ** Endoglin/Cr excluded from multivariate model due to collinearity.

**Table 7:** Diagnostic usefulness of laboratory variables for diagnosing severe, moderate renal scars and borderline lesions. **a**. Diagnostic usefulness of laboratory variables for diagnosing severe and moderate renal scars in the scintigraphy (*n* = 63). **b**. Diagnostic usefulness of laboratory variables for diagnosing renal borderline lesions in the scintigraphy (*n* = 18).

Variables	AUC (95%CI)	*p*	Cut-Off	Sensitivity	Specificity
				(%)	(%)
**a**					
**Laboratory data**					
Serum Cr (mg/dL)	0.503 (0.400–0.606)	0.958	0.40	55.0	52.4
Cystatin C (mg/L)	0.624 (0.525–0.723)	0.014	0.92	47.6	74.6
GFR (mL/min/1.73 m^2^)	0.678 (0.583–0.772)	<0.001	104.90	75.0	55.6
ACR (mg/g)	0.647 (0.550–0.744)	0.003	16.40	56.5	68.3
**Urinary biomarkers**					
TGF-β1 (ng/mL)	0.630 (0.526–0.733)	0.014	0.052	53.3	79.4
Endoglin (ng/mL)	0.579 (0.475–0.684)	0.136	11.825	38.3	87.3
Periostin (ng/mL)	0.849 (0.780–0.918)	<0.001	0.053	84.1	75.0
Cytokeratin-18 (ng/mL)	0.611 (0.511–0.712)	0.030	0.292	74.6	50.0
TGF-β1/Cr (ng/mg)	0.514 (0.410–0.618)	0.791	0.092	60.0	50.8
Endoglin/Cr (ng/mg)	0.527 (0.423–0.632)	0.606	6.794	87.5	23.8
Periostin/Cr (ng/mg)	0.810 (0.731–0.888)	<0.001	0.131	84.1	70.0
Cytokeratin-18/Cr (ng/mg)	0.580 (0.479–0.682)	0.121	0.758	60.3	58.3
**b**					
**Laboratory data**					
Serum Cr (mg/dL)	0.625 (0.473–0.778)	0.108	0.50	55.6	66.7
Cystatin C (mg/L)	0.645 (0.502–0.789)	0.047	0.80	83.3	49.2
GFR (mL/min/1.73 m^2^)	0.669 (0.533–0.804)	0.015	118.40	50.0	83.3
ACR (mg/g)	0.508 (0.341–0.676)	0.922	4.51	81.7	33.3
**Urinary biomarkers**					
TGF-β1 (ng/mL)	0.566 (0.426–0.706)	0.358	0.055	46.7	72.2
Endoglin (ng/mL)	0.661 (0.544–0.779)	0.007	10.444	48.3	94.4
Periostin (ng/mL)	0.777 (0.642–0.911)	<0.001	0.086	66.7	86.0
Cytokeratin-18 (ng/mL)	0.626 (0.480–0.771)	0.090	0.397	61.1	70.0
TGF-β1/Cr (ng/mg)	0.553 (0.396–0.710)	0.506	0.043	73.3	55.6
Endoglin/Cr (ng/mg)	0.655 (0.491–0.818)	0.064	5.496	92.9	44.4
Periostin/Cr (ng/mg)	0.658 (0.530–0.785)	0.015	0.046	94.4	38.3
Cytokeratin-18/Cr (ng/mg)	0.532 (0.377–0.688)	0.682	0.493	56.7	61.1

Cr—creatinine; GFR—estimated glomerular filtration rate; ACR—urinary albumin/creatinine ratio; TGF-β1—transforming growth factor-β1.

## Data Availability

The data analyzed in this study are available from the corresponding author on reasonable request.
